# *Escherichia coli* *γ*-carbonic anhydrase: characterisation and effects of simple aromatic/heterocyclic sulphonamide inhibitors

**DOI:** 10.1080/14756366.2020.1800670

**Published:** 2020-08-04

**Authors:** Sonia Del Prete, Silvia Bua, Claudiu T. Supuran, Clemente Capasso

**Affiliations:** a Department of Biology, Agriculture and Food Sciences, CNR, Institute of Biosciences and Bioresources, Napoli, Italy; b Section of Pharmaceutical and Nutraceutical Sciences, Department of NEUROFARBA, University of Florence, Firenze, Italy

**Keywords:** Carbonic anhydrase, sulphonamides, inhibitors, antibacterials, *Escherichia coli*

## Abstract

Carbonic anhydrases (CAs, EC 4.2.1.1) are ubiquitous metalloenzymes involved in biosynthetic processes, transport, supply, and balance of CO_2_/HCO_3_^-^ into the cell. In Bacteria, CAs avoid the depletion of the dissolved CO_2_/HCO_3_^-^ from the cell, providing them to the central metabolism that is compromised without the CA activity. The involvement of CAs in the survival, pathogenicity, and virulence of several bacterial pathogenic species is recent. Here, we report the kinetic properties of the recombinant *γ*-CA (EcoCA*γ*) encoded in the genome of *Escherichia coli*. EcoCA*γ* is an excellent catalyst for the physiological CO_2_ hydration reaction to bicarbonate and protons, with a k_cat_ of 5.7 × 10^5^ s^−1^ and k_cat_/K_M_ of 6.9 × 10^6^ M^−1^ s^−1^. The EcoCA*γ* inhibition profile with a broad series of known CA inhibitors, the substituted benzene-sulphonamides, and clinically licenced drugs was explored. Benzolamide showed a K_I_ lower than 100 nM. Our study reinforces the hypothesis that the synthesis of new drugs capable of interfering selectively with the bacterial CA activity, avoiding the inhibition of the human *α* -CAs, is achievable and may lead to novel antibacterials.

## 1. Introduction

Nature developed a fascinating system for recycling CO_2_^1^^,^^2^_._ Plant, algae, and photosynthetic prokaryotes through the photosynthetic process can convert light energy into chemical energy^1^. The last is stored in the carbohydrates, and the carbon dioxide (CO_2_) is fixed into biomass. In the aerobic environment, carbon from the biomass returns to the atmosphere by the action of O_2_-requiring decomposers, such as bacteria and fungi^3^, while, in an environment characterised by the absence of oxygen, the anaerobic microbes decompose the biomass releasing methane and CO_2_^4^_._ The carbon cycle is essential for the life on the Earth since carbon is a critical component in controlling the planet temperature, a crucial food ingredient for sustaining the entire living organisms, and, finally, an energy source for driving the global economy^5^. A necessary enzyme involved in the carbon cycle, which has the function to enhance the photosynthesis via a mechanism that concentrates and supplies CO_2_ up to 1000-fold close to the ribulose-1,5-bisphosphate carboxylase (RuBisCO), is the carbonic anhydrase (CA, EC 4.2.1.1) ^6^. Several CA-classes have been identified, such as α, β, γ, δ, ζ, η, θ, and ι^7–9^. The eight CA-classes, showing a low sequence similarity, different folds and structures, are considered phylogenetically distinct. This exceptional sequence and structural divergence evolved, reflecting a convergent evolution of the CA-classes since they target a common substrate, the CO_2,_ and catalyse the same reaction, the simple but physiologically crucial reaction of carbon dioxide hydration/dehydration to bicarbonate and protons (CO_2_ + H_2_O ⇄ HCO_3_^-^ + H^+^)^10–17^. The CO_2_ hydratase reaction is catalysed at very high rates, with a pseudo-first-order kinetic constant (k_cat_) ranging from 10^4^ to 10^6^ s^−1^ (18, 19), which is about 67,000–7,000,000 times higher than the uncatalyzed reaction with a k_cat_=0.15 s^−1^^18^^,^^19^^.^ In addition to the involvement in photosynthetic processes, CAs accomplish the transport, supply, and balance of CO_2_ and bicarbonate into the cell, but also pH homeostasis, secretion of electrolytes, and participate in several biosynthetic processes^20^^,^^21^. The homeostasis of H^+^ and CO_2_/HCO_3_^-^ is involved in many physiological and pathological conditions^18^^,^^19^^,^^22–26^. In Bacteria, for example, the primary CA function is to avoid the depletion of the dissolved inorganic carbon (CO_2_/HCO_3_^-^) from the cell, providing them quickly to the central metabolism, which might be compromised without the CA activity^7^^,^^8^^,^^16^^,^^27–29^. A charming example is represented by the *β*-CA (CynT) encoded by the genome of the bacterium *Escherichia coli*, a Gram-negative bacterium typically colonising the lower intestine of warm-blooded organisms^30–32^. The *E. coli* cyn operon contains three genes, CynT, CynS, and CynX encoding for a *β*-CA, a cyanase, and an unknown protein, respectively^33^. CynT catalysing the CO_2_ hydration produces HCO_3_^-^, whose depletion from the bacterial cells is avoided since the cyanase uses it as a substrate to produce ammonia and CO_2_^33–35^_._

Numerous examples support the importance of CA activity in the growth of bacteria. For example, the deletion of a gene encoding for the *β*-CA from *Ralstonia eutropha* allowed the heterotrophic growth of the mutant only at an elevated concentration of CO_2_ compared to wild-type^36^. In *E. coli*, a second *β*-CA, CynT2, is essential for the growth of the microorganism at atmospheric pCO_2_^37^^,^^38^_._ The lost of CA genes in some Proteobacteria, such as those belonging to the genera Buchnera and Rickettsia, determined their adaptation only in high-CO_2_ niches^39^. More interesting, are the discovery of the involvement of CAs in the survival, pathogenicity, and virulence of several species of human pathogens, such as *Helicobacter pylori*^40–42^*, Vibrio cholerae*^43^*, Brucella suis*^44–47^*, Salmonella enterica*^48^, and *Pseudomonas aeruginosa*^49^ among others.

In this context, we focalised our interest in the inhibition profile of the CAs encoded by the genome of *E. coli*, a bacteria generally coexisting in a mutually beneficial state with the host^50^. In some cases, it may become a severe pathogen^51–53^ or can cause diseases if the host defences are weakened^54^. The *E. coli* genome encodes for *β*-CAs (Cyn T and CynT2), *γ*- and ι-CAs. Here, we cloned, overexpressed, and purified the *γ*-CA (EcoCA*γ*) enzyme. The recombinant EcoCA*γ* was subjected to the investigation of its kinetic properties since only the three-dimensional structure was determined in 2012^55^. Besides, we explored the EcoCA*γ* inhibition profile with a broad series of substituted benzene-sulphonamides and clinically licenced drugs, which generally inhibit the CAs in the nanomolar range^56–58^. The EcoCA*γ* inhibition profile was compared with those obtained for the two human isoforms (hCA I and hCA II) with the prospect of gaining new scientific knowledge in the design of potentially new inhibitors capable of blocking efficiently and selectively the CA activity encoded by the pathogenic microbes.

## 2. Materials and methods

### 2.1. Chemicals and instruments

All the chemicals used in this study were of reagent grade and purchased from Sigma. The Affinity column (His-Trap FF) and the AKTA-Prime purification system were bought from GE Healthcare. The SX20 Stopped-Flow and SDS–PAGE and Western-Blot apparatus were obtained by AppliedPhotophysics and BioRAD, respectively.

### 2.2. *Cloning, expression and purification of the recombinant E.coli* *γ*-CA

The synthetic *E. coli* gene encoding for the EcoCA*γ* (Accession number: WP_009008373.1) was synthesised by the Invitrogen GeneArt (ThermoFisher Scientific), a company specialised in gene synthesis, and cloned into the expression vector pET100D-Topo/*γ*-CA. Briefly, the gene was designed to produce the recombinant EcoCA*γ* as fusion proteins with a tag containing nucleotides encoding for six histidines (His-Tag) at the amino terminus of neosynthetized recombinant protein. Competent *E. coli* BL21 (DE3) codon plus cells (Agilent) were transformed as described by Del Prete *et al*. ^59^. Isopropyl β-D-1-thiogalactopyranoside (IPTG) at the concentration of 1 mM was added to the cellular culture to overexpress the recombinant EcoCA*γ*. After growth, the cells were harvested and disrupted by sonication. Cellular extract was purified using a nickel affinity column (His-Trap FF), which allows the interaction between the matrix functionalised with Ni^2+^ ions and the His-Tag at the N-terminus of the protein. The HisTrap column (1 ml) was equilibrated with 20 ml equilibration buffer (50 mM Tris, 20 mM imidazole and 150 mM sodium chloride, pH 7.5) at 1 ml/min. The supernatant from the cellular lysate was loaded onto the column at 1 ml/min, and eluted from the column by fluxing imidazole (300 mM) at a flow of 0.5 ml/min in a buffer composed of 50 mM Tris and 300 mM sodium chloride, pH 7.5. The recovered EcoCA*γ* was 90% pure. The protein quantification was carried out by Bradford method (BioRAD) ^60^.

### 2.3. Enzyme activity for monitoring the EcoCA*γ* purification

The CA activity assay was performed as described by Capasso *et al.* ^61^. Briefly, the assay was based on the monitoring of pH variation due to the catalysed conversion of CO_2_ to bicarbonate. Bromothymol blue was used as the indicator of pH variation. The assay was performed at 0 °C and a CO_2_-satured solution was used as substrate. The enzyme activity was calculated by measuring the time required for Bromothymol blue to change from blue to yellow. This time is inversely related to the quantity of enzyme present in the sample and allows the calculation of the Wilbur-Anderson units as described previously^61^.

### 2.4. Sds-PAGE, protonography and Western-Blot

A 12% Sodium Dodecyl Sulfate-polyacrylamide gel electrophoresis (SDS-PAGE) prepared as described by Laemmli^62^ was used, loading on the gel the recovered EcoCA*γ* from the affinity column. The gel was stained with Coomassie Brilliant Blue-R. To perform the protonography, wells of 12% SDS-PAGE gel were loaded with samples mixed with loading buffer not containing 2-mercaptoethanol and not subjected to boiling, in order to avoid protein denaturation. The gel was run at 150 V until the dye front ran off the gel. Following the electrophoresis, the 12% SDS-PAGE gel was subject to protonography to detect the yellows bands due to the hydratase activity on the gel as described previously^63–66^. In addition, EcoCA*γ* was transferred to a PVDF (polyvinylidene fluoride) membrane with transfer buffer (25 mM Tris, 192 mM glycine, 20% methanol) using Trans-Plot SD Cell (Bio-Rad, Hercules, CA, USA). His-Tag Western blot was carried out using the Pierce Fast Western Blot Kit (Thermo Scientific, Waltham, MA, USA). Blotted membrane was placed in the wash blot solution Fast Western 1 Wash Buffer to remove transfer buffer. Primary Antibody Working Dilution was added to the blot and incubated for 30 min at room temperature (RT) with shaking. Invitrogen anti-His antibody (1:10000) was used. Afterwards, the blot was removed from the primary antibody solution and incubated for 10 min with the FastWestern Optimised HRP ReagentWorking Dilution. Subsequently, the membrane was washed two times in about 20 ml of FastWestern 1Wash Buffer. Finally, the membrane was incubated with the detection reagent working solution and incubated for 1 min, at room temperature, and then developed with X-ray film.

### 2.5. Kinetic parameters and inhibition constants

The CO_2_ hydration activity performed by the EcoCA*γ* was monitored using an Applied Photophysics stopped-flow instrument^67^. Phenol red (at a concentration of 0.2 mM) was used as indicator, working at the absorbance maximum of 557 nm, with 20 mM TRIS (pH 8.3) as buffer, and 20 mM NaClO_4_ (for maintaining constant the ionic strength), following the initial rates of the CA-catalysed CO_2_ hydration reaction for a period of 10–100 s. To determine the kinetic parameters by Lineweaver-Burk plots and the inhibition constants, a concentration of CO_2_ between 1.7 to 17 mM was used. At least six measurements of the original 5–10% reaction were used to assess the initial velocity for each inhibitor. The uncatalyzed rates were identically determined and detracted from the total observed rates. Stock inhibitor solutions (10–100 mM) were prepared in distilled-deionized water and dilutions up to 0.01 mM were done with the buffer test. Inhibitor and enzyme solutions were preincubated together for 15 min at room temperature prior to assay, in order to allow for the formation of the E-I complex or for the eventual active site mediated hydrolysis of the inhibitor. The inhibition constants were obtained by non-linear least-squares methods using PRISM 6 and the Cheng-Prusoff equation, as reported earlier^12^^,^^14^^,^^68^, and represent the mean from at least three different determinations. h CAI and hCA II were recombinant enzymes obtained in-house.

## 3. Results and discussion

### 3.1. Primary structure analysis

**Figure 1.**
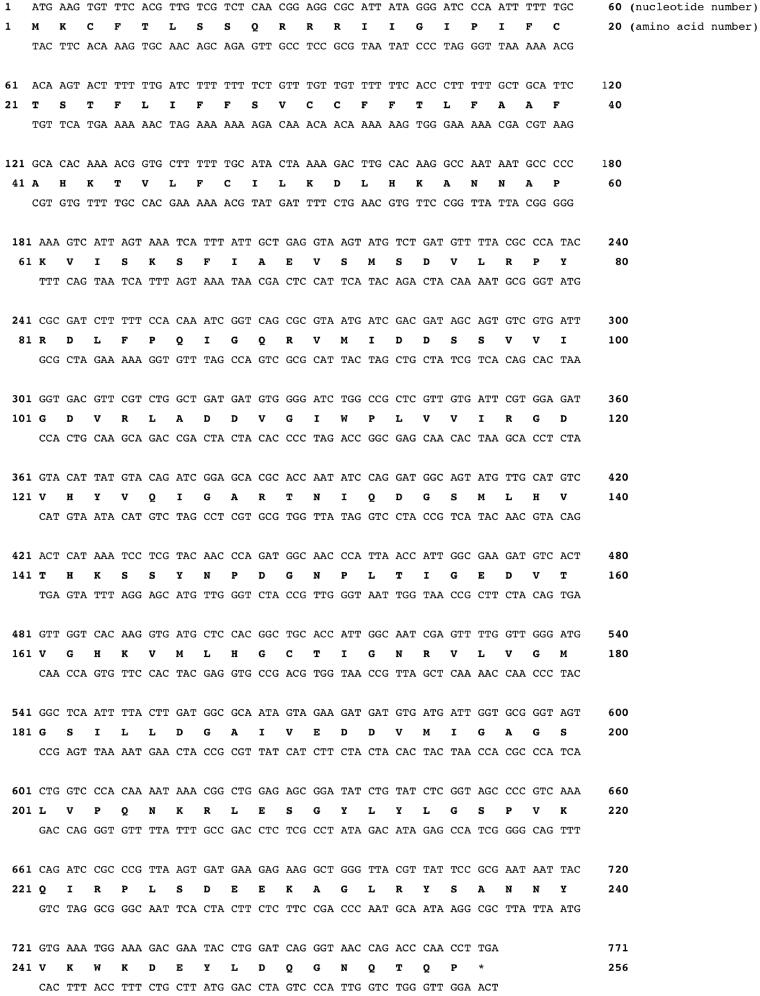
Nucleotide and translated amino acid sequences of the EcoCA*γ*. The amino acid residues and the open reading frame are indicated by capital letters, in bold and not in bold, respectively, *, indicate the stop codon.

**Figure 2.**
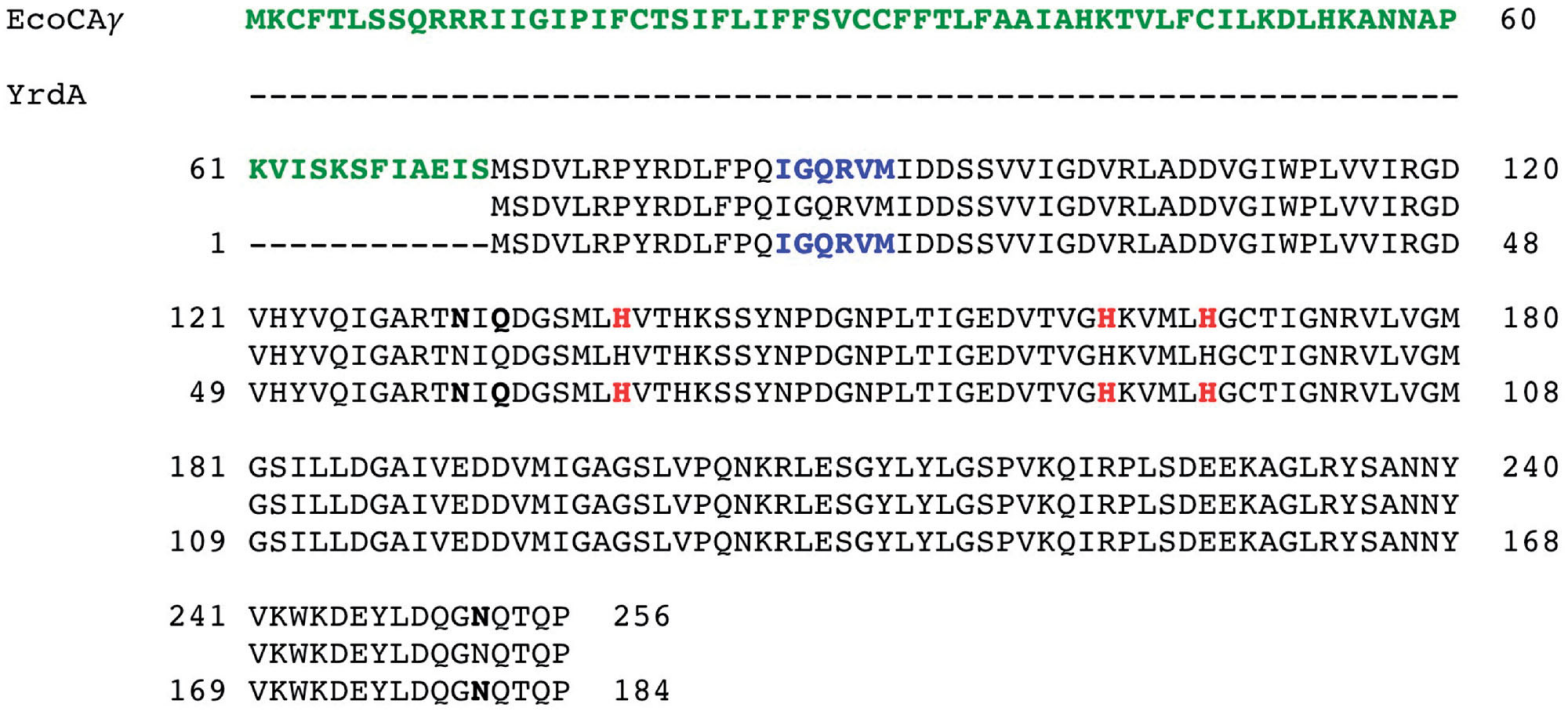
Pairwise comparison of EcoCA*γ* amino acid sequence with the YrdA polypeptide chain. The pairwise alignment was performed with the programme Blast Global Align. The accession numbers of the aligned sequences are WP_009008373.1 (EcoCA*γ*) and P0A9W9 (YrdA). Legend: The extra 72 amino acid residues are in green bold; the identical amino acid residues are between the two aligned sequences; the amino acid residues of a typically repeated hexapeptide are reported in bold blue; the three histidines coordinating the metal ion are in red bold; the catalytically relevant residues, which participate in a network of hydrogen bonds with the catalytic water molecule, are represented in black bold; a hyphen shows gaps.

Because the genome of *Escherichia coli* was full sequenced, it was designed and polymerised the synthetic gene encoding for the EcoCA*γ* polypeptide chain. The sequence of the synthetic gene and its corresponding protein are shown in Figure 1. The EcoCA*γ* nucleotide sequence consists of an open reading frame of 771 nucleotides, encoding for a polypeptide chain of 256 amino acid residues (Figure 1). The EcoCA*γ* was aligned with the YrdA amino acid sequence, which corresponded to the *γ*-CA crystallised in 2012 (55). As shown in Figure 2, EcoCA*γ* has the same polypeptide chain of the YrdA protein, except for the presence of 72 additional amino acids at the N-terminus. In bacteria genes are often found in a cluster on the chromosome, which are under control of a single promoter^69^. This gene organisation is known as an operon. Thus, it is possible that the additional 72 residues belong to a different protein encoded by the *E. coli* operon, which includes the gene encoding for the E.coli *γ*-CA, too. As described in the literature, *γ*-CA is a homotrimer with three zinc-containing active sites located at the interfaces between two monomers^55^. Each monomer is structurally characterised by a tandemly-repeated hexapeptide, which mostly shows an aliphatic residue, usually Ile, Val, or Leu, at the first position, a well-conserved residue is glycine at position two, and a residue Ala, Ser, Cys, Val, Thr, or Asn at position five (Figure 2) ^70^. This repeated hexapeptide is essential for the left-hand fold of the trimeric *β*-helix structures, which contradistinguish the canonical *γ*-CAs and putative acetyltransferases/acyltransferases^55^. YrdA was crystallised without the extra 72 residues at the N-terminus because the authors considered only the polypeptide chain with the conserved hexapeptide-repeat motifs (182 amino acid residues), which generally typify all the *γ*-CA sequences (Figure 2).

Next paragraphs report 1) the heterologous expression and purification of the EcoCA*γ* full amino acid sequence (containing the extra 72 residues at the N-terminus of the polypeptide chain); 2) the determination of the kinetic parameters of EcoCA*γ* using the stopped-flow technique; and 3) the inhibition profile of EcoCA*γ* with a broad range of small molecules, which generally inactivate this class of enzyme.

### 3.2. Heterologous expression and purification

**Figure 3.**
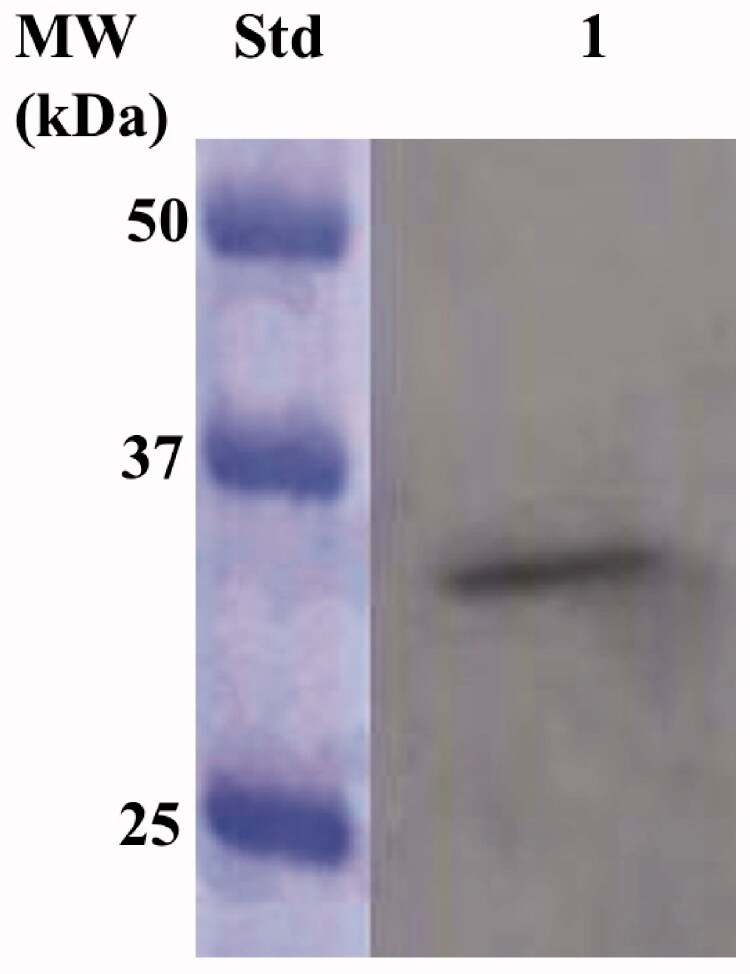
Western blot analysis performed on the supernatant coming from the *E. coli* cellular extract obtained after the sonication and centrifugation. Lane STD, molecular markers, M.W. starting from the top: 50 kDa, 37 kDa, and 25 kDa; lane 1, overexpressed chimeric EcoCA*γ*.

IPTG ((Isopropyl β-D-1-thiogalactopyranoside) induction of *E. coli* BL21 (DE3) cells transformed with the expression plasmid containing the complete EcoCA*γ* gene resulted in the production of the recombinant *γ*-CA as a chimeric polypeptide chain obtained by the fusion of the N-terminus of the EcoCA*γ* protein to the C-terminus of a soluble peptide (about 4.0 kDa) containing six histidines (His-Tag). This strategy has been adopted to improve the solubility, purification, and detection of the recombinant EcoCA*γ* expressed in its complete form (plus the extra 72 residues at the N-terminus). After the sonication and centrifugation, most of the CA activity was recovered in the soluble fraction of the *E. coli* cell extract. The expression of the chimeric EcoCA*γ* was confirmed by Western Blot (WB) analysis using an anti-His-Tag antibody (Figure 3). As expected, analysing the *E. coli* cellular extract, the specific antibody for the His-Tag tail recognised the fusion protein as a band with a molecular weight of about 33.0 kDa. A subunit molecular mass of 32.4 kDa was calculated on the amino acid sequence translated from the chimeric gene encoding for the chimeric EcoCA*γ*.

**Figure 4.**
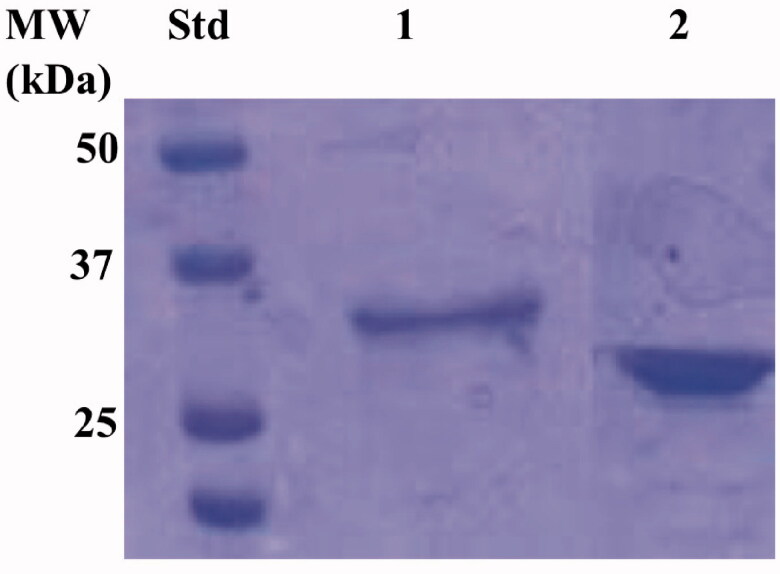
Electropherogram of the SDS-PSAGE carried out on the recombinant chimeric EcoCA*γ*. Legend: lane STD, molecular markers, M.W. starting from the top: 50 kDa, 37 kDa, and 25 kDa; lane 1, chimeric EcoCA*γ*; Lanes 2 commercial bovine CA (bCA) used as control. The band at a molecular weight of about 33.0 kDa represented the chimeric EcoCA*γ* purified by affinity chromatography.

The recombinant enzyme was purified to homogeneity using the affinity chromatography, as demonstrated by the SDS-Page analysis (Figure 4). As a result, the electropherogram profile of EcoCA*γ* showed a band at the same position identified in the western-blot analysis (33.0 kDa) under reducing conditions (Figure 4).

**Figure 5.**
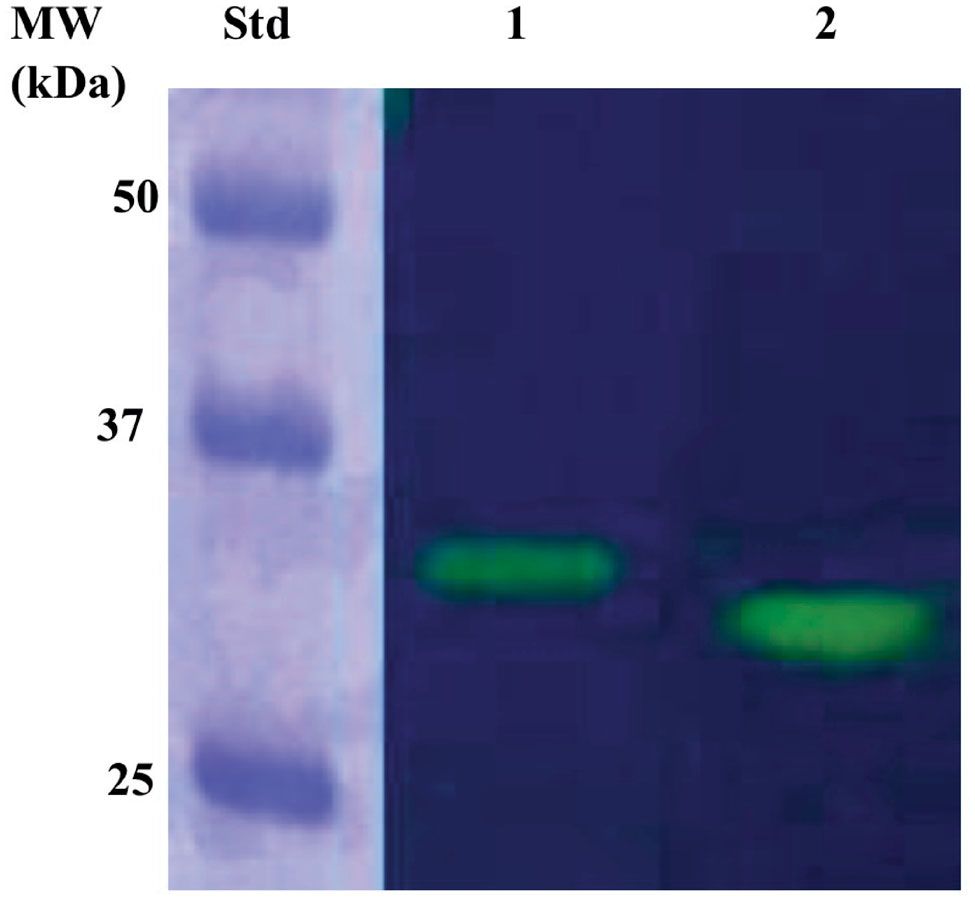
Protonogram of the EcoCA*γ* eluted from the affinity resin. The yellow band on the protonogram corresponds to the enzyme activity responsible for the drop of pH from 8.2 to the transition point of the dye in the control buffer (pH 6.8), due to the hydratase activity. Lane STD, molecular markers starting from the top: 50 kDa, 37 kDa, and 25 kDa); Lane 2, purified EcoCA*γ*; Lane 3, commercial bovine CA (bCA) used as a positive control.

The full sequence of the recombinant EcoCA*γ* was also subjected to protonography, a powerful and elegant technique able to reveal, as a yellow band on the polyacrylamide gel, the production of ions (H^+^) developed during the CO_2_ hydration reaction^63^^,^^65^. The protonography analysis demonstrated that the complete EcoCA*γ* polypeptide chain was catalytically active. The protonogram showed a single hydratase activity band on the gel with a molecular weight of about 33.0 kDa, corresponding to the monomeric form of the chimeric EcoCA*γ* (Figure 5). This was not a surprise since the protonography analysis requires the elimination of SDS at the end of the electrophoretic run. Even if all the *γ*-CAs are active as trimers, the yellow band appeared in the position of the inactive monomeric form because the SDS purging leads to the rearrangement of the *γ*-CA monomers in the gel. As a result, EcoCA*γ* correctly refolded and generated the active trimeric forms of the *γ*-CA at the position of the monomer. This is described for other eukaryotic and prokaryotic CA classes, too^63^

### 3.3. Determination of the kinetic parameters using the stopped-flow technique

The CO_2_ hydratase activity of the soluble enzyme and its kinetic constants were determined using the stopped-flow technique (Table 1). These results were compared with the kinetic parameters of the two mammalian *α*-CA isoforms (h CAI and h CAII), as well as with the β-CA from the same microorganism and the *α*-, β-, and γ- CAs from *Vibrio cholerae* (Table 1).

**Table 1.** Kinetic parameters for the CO_2_ hydration reaction catalysed by the α-, β-, and γ-class CA enzymes: hCA I and II (α-class CAs), and VchCAα at 20 °C and pH 7.5 in 10 mM HEPES buffer; VchCAβ, VchCAγ; CynT2 and EcoCA*γ* determined at 20 °C, pH 8.3 in 20 mM TRIS buffer and 20 mM NaClO4. Inhibition data with the clinically used sulphonamide AZA (5-acetamido-1,3,4-thiadiazole-2-sulphonamide) are also provided.

Organism	Acronym	Class	k_cat_ (s^−1^)	k_cat_/K_m_ (M^−1^ x s^−1^)	K_I_ (acetazolamide) (nM)
*Homo sapiens*^a^	hCA I	α	2.0 x 10^5^	5.0 x 10^7^	250
	hCA II	α	1.4 x 10^6^	1.5 x 10^8^	12
*Vibrio cholerae*^a^	VchCAα	α	8.2 x 10^5^	7.0 x 10^7^	6.8
	VchCAβ	β	3.3 x 10^5^	4.1 x 10^7^	451
	VchCAγ	γ	7.3 x 10^5^	6.4 x 10^7^	473
*Escherichia coli*	CynT2^b^	β	5.3 x 10^5^	4.1 x 10^7^	227
	EcoCA*γ*	γ	5.7 x 10^5^	6.9 x 10^6^	248

Mean from 3 different assays by a stopped flow technique (errors were in the range of ±5–10% of the reported values). ^a^From reference (14); ^b^From reference (50).

As shown in Table 1, EcoCA*γ* is an excellent catalyst for the physiological CO_2_ hydratase reaction to bicarbonate and protons, with a k_cat_ of 5.7 × 10^5^ s^−1^ and catalytic efficiency (k_cat_/K_M_) of 6.9 × 10^6^ M^−1^ s^−1^. EcoCA*γ* k_cat_ was similar to those obtained for other bacterial CAs belonging to the β-or *γ*- classes, as well as for the human isoform hCA I. Interestingly, the catalytic efficiency of EcoCA*γ* resulted to be two orders of magnitude lower with respect to that of hCA II and one order with respect to VchCAγ (same class of EcoCA*γ*) and the other enzymes reported in Table 1. The investigation of the kinetic properties of the CAs is important because even if these enzymes belong to the same or different CA-classes the steric hindrance of the amino acid residues surrounding the catalytic pocket is responsible for the increase/decrease of the parameters related to the affinity of the enzyme for the substrate (K_M_). EcoCA*γ* was also inhibited by the sulphonamide acetazolamide (K_I_ = 248 nM), a well-known pharmacological CA inhibitor (Table 1). The acetazolamide resulted to be a very sensitive inhibitor of the human isoform h CAII (K_I_=12 nM) and the *α*-CA from *Vibrio cholerae* (K_I_=6.8 nM), wheras for the other CAs reported in Table 1 it was less effective, with K_I_s in the range of 227–473 nM. Thus, the K_I_ variation can be attributed to the interaction and steric hindrance of the amino acid residues of the catalytic pocket interacting with the inhibitor. The structural differences, which affect the CA-classes or CAs belonging to the same class, highlight the possibility of designing specific and selective inhibitors for this superfamily of enzymes.

### 3.4. Sulphonamide inhibition profile

**Figure 6.**
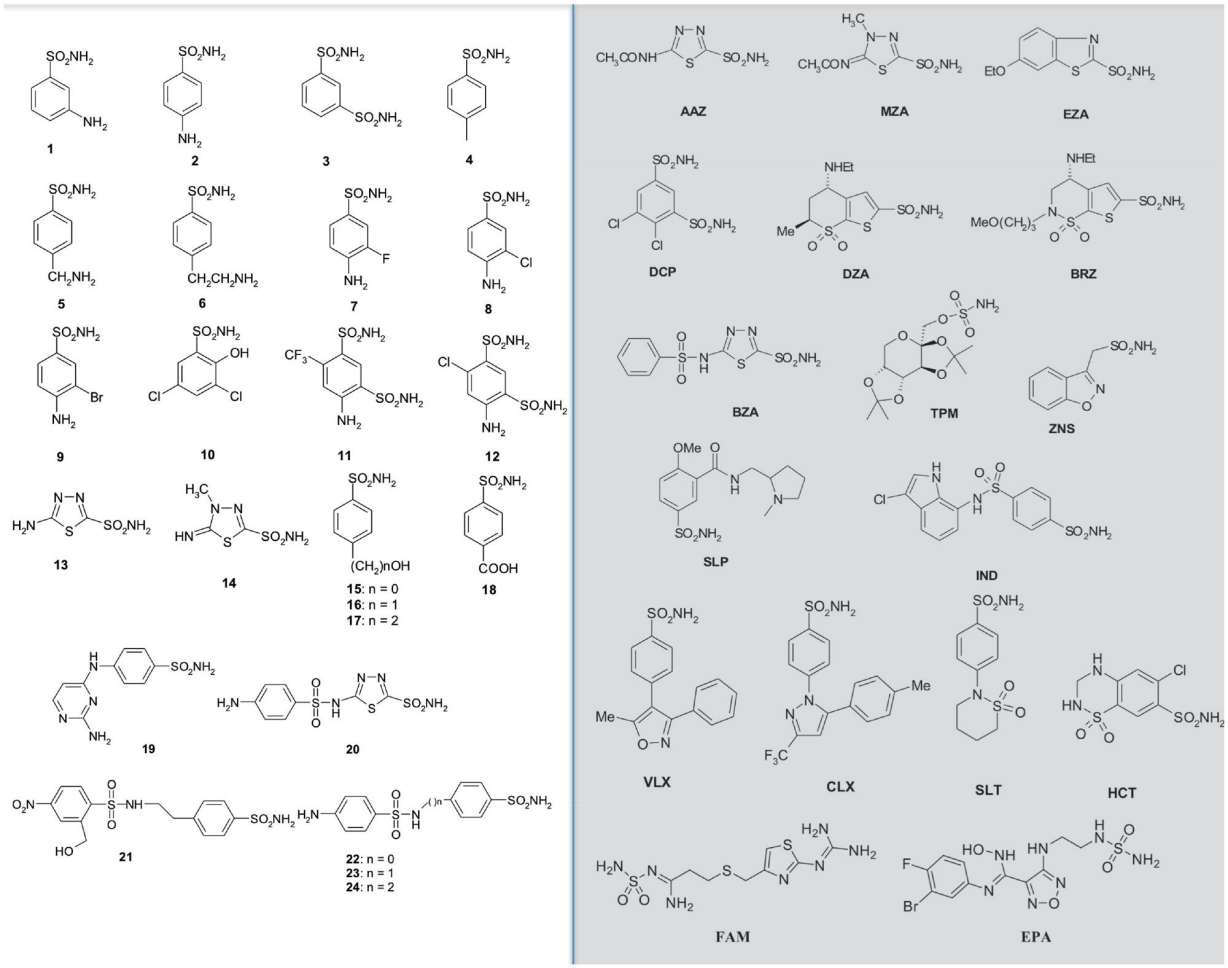
The 42 compounds used to study the EcoCA*γ* inhibition profile. Forty-one sulphonamides and one sulfamate (TPM) were exploited. On the left, the series 1–24; on the right and grey, the clinically used drugs.

Among the CAIs, a library of 42 compounds, **1–24** and **AAZ-EPA,** represent an important group of simple aromatic/heterocyclic sulphonamides (including one sulfamate), which are able to inhibit the CA (Figure 6).

The series **AAZ-EPA** includes licenced drugs used for the following clinical treatments: glaucoma, epilepsy, idiopathic intracranial hypertension, diuretic, duodenal ulcers, migraine, Parkinson’s disease, obesity, cancer, osteoarthritis, rheumatoid arthritis, diet, etc.^56–58^. Most of the sulphonamides bind in a tetrahedral geometry to the Zn(II) ion in the deprotonated state, establishing with the amino acid residues of the catalytic site an extended network of hydrogen bonds^18^^,^^19^^,^^22^, as well as the aromatic/heterocyclic part of the inhibitor interacts with the hydrophilic and hydrophobic residues of the catalytic cavity^18^^,^^19^.

Since CAs are considered a valuable target for impairing the microbe vitality or their virulence, the *in vitro* exploration of the EcoCA*γ* inhibition profile with such inhibitors is crucial for obtaining potent and selective families of new pharmacological agents. New drugs are needed considering that the emergence arisen from the resistance to the existing antimicrobial medicines is one of the most severe problems afflicting the human community. Table 2 reports the inhibition profile of EcoCA*γ*, which was compared with the inhibitory behaviour of hCA I, hCA II, and VchCAγ reported earlier by our group^17^^,^^56^^,^^71^. From the data of Table 2, the following can be observed:

**Table 2.** Inhibition of hCA I, hCA II, EcoCA*γ* and VchCAγ with sulphonamides **1–24** and the clinically used drugs **AAZ-EPA**.

	K_I_*(nM)			
Inhibitor	hCA I^a^	hCA II^a^	EcoCA*γ*	VchCAγ ^a^
1	28000	300	314	672
2	25000	240	193	95
3	79	8	246	94
4	78500	320	221	76
5	25000	170	160	81
6	21000	160	622	69
7	8300	60	605	74
8	9800	110	671	74
9	6500	40	718	95
10	7300	54	2577	544
11	5800	63	1779	87
12	8400	75	1953	563
13	8600	60	197	66
14	9300	19	712	70
15	5500	80	1013	88
16	9500	94	4238	556
17	21000	125	1975	6223
18	164	46	2064	5100
19	109	33	1894	4153
20	6	2	883	5570
21	69	11	819	764
22	164	46	3501	902
23	109	33	4045	273
24	95	30	4262	73
AAZ	250	12	248	473
MZA	50	14	921	494
EZA	25	8	5538	85
DCP	1200	38	889	1230
DZA	50000	9	2007	87
BRZ	45000	3	4842	93
BZA	15	9	94	78
TPM	250	10	648	69
ZNS	56	35	755	725
SLP	1200	40	914	78
IND	31	15	387	91
VLX	54000	43	891	817
CLX	50000	21	944	834
SLT	374	9	446	464
SAC	18540	5959	4903	550
HCT	328	290	3643	500
FAM	922	58	274	–
EPA	8262	917	744	–

* Errors in the range of 5–10% of the reported data, from 3 different assays (data not shown). ^a^Human recombinant isozymes and *Vibro enzyme* stopped flow CO_2_ hydrase assay method from ref.^14^; –, not detected.

1. Among the sulphonamides and sulfamate used to determine the EcoCA*γ* inhibition profile, only one inhibitor resulted in a K_I_ lower than 100 nM. This is the case of the benzolamide (**BZA**) whit a K_I_ = 94 nM. Generally, **BZA** is clinically used in the treatment of glaucoma. The two human isoforms, hCA I and hCA II, resulted very sensitive to such inhibitor with a K_I_ of 15 and 9 nM, respectively. The *Vibrio* enzyme was inhibited with a K_I_ = 78 nM. *V. cholerae* enzyme showed a large number of nanomolar inhibitors with a K_I_ below 100 nM, such as compounds **2**, **3**, **4**, **5**, **6**, **7**, **8**, **9**, **11**, **13**, **14**, **15**, **24**, **EZA**, **DZA**, **BRZ**, **BZA**, the sulfamate **TMP**, **SLP,** and **IND**. Except for BZA, these inhibitors inhibited EcoCA*γ* with K_Is_ in the range 193–1013 nM for the series **1–24**, and K_Is_ of 387-5538 nM for the proposed licenced drugs. The analysis of the three-dimensional structures of EcoCA*γ* and VchCA*γ* (not available at this moment) will allow the identification of the structural factors responsible for the K_I_ variations, and thus the possibility to design efficient and selective inhibitors of the bacterial enzymes.
2. Most of the inhibitors considered in the present study were moderate inhibitors of EcoCA*γ* with K_Is_ in the range 160–944 nM, such as compounds from **1** to **9**, **13**, **14**, **20**, **21**, **AZA**, **MZA**, **DCP**, **TMP**, **ZNS**, **SLP**, **IND**, **VLX**, **CLX**, **SLT**, **FAM,** and **EPA**. It is important to note that some of these inhibitors were very sensitive versus the human isoform h CAII and harmful inhibitors for the human isoform h CA I (KI = 6.6 – 78.5 µM). The zonisamide (**ZNS**) an aliphatic primary sulphonamide, was also a very weak inhibitor for the bacterial enzymes (K_Is_= 725 nM) but effective towards the human isoenzymes (K_Is_= 35–56 nM). The K_I_ differences reported in the Table 2 evidenced that the inhibition pattern is almost different also between the human isoenzymes corroborating the idea that the comparison of the inhibition profile represents a potent tool for developing new specific inhibitors. For example, it is possible to tune and/or design specific inhibitors for the isoforms based on their structural differences, even if the isoenzymes show a high percentage of amino acid sequence identity.
3. Several substituted benzene-sulphonamides, such as **10**, **11**, **12**, **15**, **16**, **17**, **18**, **19**, **22**, **23**, **24**, **EZA**, **DZA**, and **BRZ**, were ineffective week inhibitors of the EcoCA*γ*, showing K_Is_ in the range of 1.0–5.5 µM. Moreover, most of these inhibitors, such as the compounds **11**, **24**, **EZA**, **DZA**, and **BRZ**, inhibited the Vibrio enzyme (VchCA*γ*) with K_Is_ =73–93 nM.

The behaviour of EcoCA*γ* is somewhat challenging to explain observing the different inhibition profiles and comparing them to the orthologous VchCA*γ* and the two human isoforms (hCA I and hCA II). EcoCA*γ* seems less or not inhibited by most of the substituted benzene-sulphonamides and clinically licenced drugs. However, considering that also the two human isoforms showed a sulphonamide inhibition pattern different from each other, as well as from the bacterial enzymes, it is reasonable to support the thesis concerning the synthesis of new drugs capable of interfering selectively with EcoCA*γ* or VchCA*γ* activity, avoiding the inhibition of the human CAs (*α*-class enzymes).

## 4. Conclusions

*Escherichia coli* is an opportunistic pathogen typically colonising the lower intestine of warm-blooded organisms^51–54^. In the present manuscript, we focussed our interest on the EcoCA*γ* (*γ*-CA) encoded by the *E. coli* genome since bacterial CAs are considered a valuable target for impairing the microbe vitality or the bacterial virulence. EcoCA*γ* was cloned, expressed, purified, and investigated for its kinetic properties, the last not explored previously even if the three-dimensional structure was solved in 2012 (55). EcoCA*γ* resulted in an excellent catalyst for the physiological CO_2_ hydratase reaction to bicarbonate and protons, with a k_cat_ of 5.7 × 10^5^ s^−1^ and catalytic efficiency (k_cat_/K_M_) of 6.9 × 10^6^ M^−1^ s^−1^. A broad range of substituted benzene-sulphonamides and clinically licenced drugs were used to determine the inhibition profile of EcoCA*γ*, the ortholog enzyme VchCA*γ*, and the possible off-targets hCA I and hCA II. Among the sulphonamides and the one sulfamate used as inhibitors, only one of them resulted in a K_I_ lower than 100 nM. This is the case of the benzolamide (**BZA**) whit a K_I_= 94 nM. Generally, **BZA** is clinically used in the treatment of glaucoma. All the other inhibitors had K_Is_ > 100 nM. Surprisingly, for most of the inhibitors used, EcoCA*γ* showed 0.5 <K_is_< 0.5 µM, evidencing that enzyme form *E. coli* was less or not inhibited by most of the substituted benzene-sulphonamides and clinically licenced drugs. As a consequence, the difference in sulphonamide inhibition pattern of the two human isoforms, as well as the two orthologs bacterial enzyme fortifies the thesis that the synthesis of new drugs is capable of interfering selectively with EcoCA*γ* or VchCA*γ* activity, avoiding the inhibition of the human CAs (*α*-class enzymes).
